# Cell-free fetal DNA testing and its correlation with prenatal indications

**DOI:** 10.1186/s12884-021-04044-5

**Published:** 2021-08-24

**Authors:** Jing-wei Wang, Yong-nan Lyu, Bin Qiao, Yan Li, Yan Zhang, Pavan Kumar Dhanyamraju, Yevgeniya Bamme, Michael D. Yu, Dongqin Yang, Yong-qing Tong

**Affiliations:** 1 Department of Clinical Laboratory, Renmin Hospital of Wuhan University, 99 Ziyang Road of Wuchang District, Wuhan, 430060 China; 2 Pennsylvania State University College of Medicine and Hershey Medical center, Hershey, PA 17033 USA; 3 Jefferson University Hospital, 1025 Walnut St, Philadelphia, PA19107 USA; 4 Department of Digestive Diseases, Huashan Hospital, Fudan University, 12 Middle Wulumuqi Road, Shanghai, 200040 China

**Keywords:** cffDNA, Prenatal screening, Aneuploidy, Prenatal diagnosis, Whole-genome sequencing, Chromosomal abnormalities

## Abstract

**Background:**

The prenatal test of cell-free fetal DNA (cffDNA) is also known as noninvasive prenatal testing (NIPT) with high sensitivity and specificity. This study is to evaluate the performance of NIPT and its clinical relevance with various clinical indications.

**Methods:**

A retrospective analysis was conducted on 14,316 pregnant women with prenatal indications, including advanced maternal age (≥35 years), maternal serum screening abnormalities, the thickened nuchal translucency (≥2.5 mm) and other ultrasound abnormalities, twin pregnancy/IVF-ET pregnancy, etc. The whole-genome sequencing (WGS) of maternal plasma cffDNA was employed in this study.

**Results:**

A total of 189 (1.32%) positive NIPT cases were identified, and 113/189 (59.79%)cases were confirmed by invasive prenatal testing. Abnormal serological screening (53.14%) was the most common indication, followed by elderly pregnancy (23.02%). The positive prediction value for T21, T18, T13, sex chromosome abnormalities, other autosomal aneuploidy abnormalities, and CNV abnormalities were 91.84, 68.75,37.50, 66.67, 14.29, and 6.45%, respectively. The positive rate and the true positive rate of nuchal translucency (NT) thickening were the highest (4.17 and 3.33%), followed by the voluntary requirement group (3.49 and 1.90%) in the various prenatal screening indications. The cffDNA concentration was linearly correlated with gestational age (≥10 weeks) and the positive NIPT group’s Z-score values.

**Conclusions:**

whole-genome sequencing of cffDNA has extremely high sensitivity and specificity for T21, high sensitivity for T18, sex chromosome abnormalities, and T13. It also provides evidence for other abnormal chromosomal karyotypes (CNV and non-21/18/13 autosomal aneuploidy abnormalities). The cffDNA concentration is closely related to the gestational age and determines the specificity of NIPT. Our results highlight NIPT’s clinical significance, which is an effective prenatal screening tool for high-quality care of pregnancy.

## Highlights

- The whole-genome sequencing of cell-free fetal DNA from maternal plasma is an effective prenatal screening tool for pregnancies with various prenatal indications.
- The concentration of cffDNA was linear with gestational age and the Z-score values of the positive NIPT group.
- NIPT has a significant positive predictive value for pregnancies with prenatal indications.

## Background

The prenatal test of cell-free fetal DNA (cffDNA), also known as noninvasive prenatal testing (NIPT) is an attractive alternative to serum screenings and invasive tests owing to its high sensitivity and specificity [1–3]. Currently, NIPT is primarily used for screening high-risk pregnancies such as trisomy 21, trisomy 18, and trisomy 13. However, it may have inconsistent results with ultrasound examination and/or actual fetal chromosome composition because the plasma cffDNA is mainly derived from placental trophoblast cells. Also, many factors may cause false positive of NIPT testing, including the maternal chromosomal abnormalities, maternal tumors (Hodgkin and non-Hodgkin lymphoma, breast, ovarian and cervical cancers, etc.), vanishing twins, placenta confined placental mosaicism (CPM), even placental chimerism [4–6]. So far, NIPT has been used as a screening method but not as a diagnostic test; it cannot replace invasive diagnostic approaches such as chorionic villus sampling and amniocentesis.

The two most widely offered NIPT methodologies were single-nucleotide polymorphism (SNP), and the whole-genome sequencing (WGS) approaches. Both share comparable clinical sensitivities for detecting common aneuploidies: T21, T18, T13, and monosomy X. In the present study, we explored NIPT screening’s clinical significance as a useful prenatal screening tool for the high-quality care of pregnancy via analyzing the 14,316 plasma cffDNA WGS screening data from pregnant women in Central China. We also explored the correlation of NIPT with the prenatal indications, particularly its sensitivity and specificity in detecting trisomies 21, 18, and 13, sex chromosome aneuploidy (SCA), non-21/18/13 autosomal aneuploidy and CNV abnormalities. We also analyzed the possible causes for false-positive and false-negative NIPT results.

## Methods

### Patient information

A total of 14,316 pregnant women were included from October 2013 to November 2018 at Renmin Hospital of Wuhan University, and also the ones referred from the following 14 hospitals: Wuhan General Hospital, Jinmen Second People Hospital, Daye People’s Hospital, Daye Chinese Medicine Hospital, Qingshan District Maternal, and Child Health Hospital, Lichuan City Hospital of Traditional Chinese Medicine, Tuanfeng County People’s Hospital, Yi Du Maternal and Child Health Hospital, Lichuan Maternal and Child Health Hospital, Tianmen First People’s Hospital, Badong County People’s Hospital, Laifeng County Central Hospital, Tuanfeng County People’s Hospital, Guangshui Maternal and Child Health Hospital, and Hong’an People’s Hospital. The testing for all the recruited participants was performed at Renmin Hospital of Wuhan University. The institutional review board of Renmin Hospital of Wuhan University approved the study. Informed consent was obtained from all participants.

The participants include pregnant women with high-risk pregnancy indicators, missing maternal serum screening test or ultrasound screening opportunities, interventional surgery contraindication (infection including HBV+, HCV+, HIV+, etc., and coagulopathy, etc), and volunteers (Table 1). The high-risk pregnancies indicators included advanced maternal age (AMA, ≥35 years old), abnormal maternal serum screening (aMSS, high risk: T21 ≥ 1/270, T18 ≥ 1/350; T13 ≥ 1/100 and intermediate-risk: 1/1000 ≤ T21 < 1/270; 1/1000 ≤ T18 < 1/350; 1/1000 ≤ T13 < 1/100), the thickened nuchal translucency (NT, NT ≥ 2.5 mm) and any other reported abnormal ultrasound findings (aUS) in fetus and placenta diagnosed by the experts, twin pregnancy/IVF-ET pregnancy (Table 1). Patients with NIPT (+) patients were classified as a positive group, and the patients with both NIPT (+) and interventional prenatal diagnosis (+) were classified as a true positive group. The invasive prenatal diagnosis included the karyotype analysis or microarray analysis of chorion, amniotic fluid, and cord blood. The blood samples were collected from the peripheral venous blood of each participant.

**Table 1:** The efficacy of indications in detecting chromosomal abnormality

Clinical indications	Characteristic			Population with high risk		T21		T18		T13		SCAs	
	Total(%)	Maternal age (years)	GA at NIPT (wks)	P (TP)	PPV	P (TP)	PPV	P (TP)	PPV	P (TP)	PPV	P (TP)	PPV
**AMA (≥35 years)**	3295 (23.02)	37.39 ± 2.37	16.54 ± 2.58	63 (38)	60.32%	23 (21)	91.30%	8 (6)	75.00%	6 (2)	33.3%	15 (9)	60.00%
**aMSS**	7608 (53.14)	28.06 ± 3.52	17.40 ± 2.83	24 (11)	45.83%	5 (5)	100.00%	2 (1)	50.00%	1 (0)	0.00%	8 (5)	62.50%
**abnormally thickened NT**	120 (0.84)	27.58 ± 3.54	15.32 ± 2.17	5 (3)	60.00%	1 (1)	100.00%	2 (2)	100.00%	0 (0)	NA	1 (0)	0.00%
**aUS**	112 (0.78)	27.69 ± 3.22	22.69 ± 3.04	3 (1)	33.33%	0 (0)	NA	0 (0)	NA	0 (0)	NA	2 (1)	50.00%
**Twin /IVF-ET pregnancy**	429 (3.00)	28.81 ± 3.27	15.26 ± 2.05	6 (5)	83.33%	3 (3)	100.00%	0 (0)	NA	0 (0)	NA	3 (2)	66.67%
**Miss other screening opportunities**	5 (0.03)	25.80 ± 2.99	25.40 ± 0.49	0 (0)	0.00%	0 (0)	NA	0 (0)	NA	0 (0)	NA	0 (0)	NA
**Voluntary Testing**	2524 (17.63)	27.59 ± 3.68	16.03 ± 2.23	88 (42)	47.73%	17 (15)	88.24%	4 (2)	50.00%	9 (4)	44.44%	34 (21)	61.76%
**Interventional surgery contraindication**	14 (0.10)	27.64 ± 2.12	16.29 ± 1.39	0 (0)	0.00%	0 (0)	NA	0 (0)	NA	0 (0)	NA	0 (0)	NA
**Other risk factors**	209 (1.46)	28.44 ± 3.41	15.35 ± 2.35	0 (0)	0.00%	0 (0)	NA	0 (0)	NA	0 (0)	NA	0 (0)	NA
**Total**	14,316			189 (100)	59.79%	49 (45)	91.84%	16 (11)	68.75%	16 (6)	37.5%	63 (38)	60.32%

Data are given as mean (range) or n (%)

Others – all other combinations except the indications in the table; abnormal ultrasound findings (aUS), abnormal maternal serum screening (aMSS, high risk: T21 ≥ 1/270, T18 ≥ 1/350; T13 ≥ 1/100 and intermediate risk: 1/1000 ≤ T21 < 1/270; 1/1000 ≤ T18 < 1/350; 1/1000 ≤ T13 < 1/100), nuchal translucency (NT) (increase extent > 95), advance maternal age (AMA); interventional surgery contraindication includes infection and coagulopathy; Other risk factors group include the pregnant women with other inherited disease, chromosomal polymorphism, drugs use history during pregnancy, medical history. or mental abnormality, et al

*P* Positive, the NIPT(+), *TP* True positive, both NIPT(+) and interventional prenatal diagnosis (+), *PPV* positive predictive value, Down syndrome, *GA* gestational age, *NA* not applicable, *wks* weeks

Of the 14,316 pregnant women, 13,162 were in singletons, and 458 in twins, with the average age of 29.71 ± 5.11 ranged from16–51 years, and the gestational age 16.99 ± 2.82 weeks with a range of 9–36 weeks. The body mass index (BMI) of participants was from13.67–42.36, with an average of 22.52 ± 3.16.

### cffDNA preparation and sequencing

Maternal peripheral blood samples (5 ml) were collected in EDTA tubes, thoroughly mixed, and stored temporarily in 4 °C refrigerators. Samples were excluded if hemolysis occurred or the sample was stored beyond 8 h before plasma separation. The plasma was isolated and dispensed into 2.0 mL Eppendorf tubes by centrifugation of the blood samples at 1600×g, 4 °C, for 10 min. The resulted plasma was centrifuged again at 16,000 x g 4 °C, for another 10 min. The supernatant was carefully aliquoted with 600 μL each into new 2.0 mL Eppendorf tubes and stored at -80 °C for cffDNA extraction. The plasma cffDNA extraction was performed with NucleoMag cfDNA isolation kit (Takara, Beijing, China) following the manufacturer’s manual. Fetal Chromosome Aneuploid (T21, T18, and T13) Detection Kit (CapitalBio Corporation, Beijing, China) was used for library construction/quality control and library amplification. The resulted libraries were sequenced using the BioelectronSeq 4000 Semiconductor Sequencing System (CapitalBio Corporation, Beijing, China) following the manufacturer’s instruction.

### Bioinformatic analysis

The obtained readings were aligned to the human genomic reference sequences (hg19) using the BWA algorithm. On average, the depth of sequencing of this NIPT method for each sample was about 0.1× sequencing depth. An in-house Perl script for FLAG field in the alignment file was used to filter the unmapped reads or those with multiple primary alignment records. An integrated three-step process, including LOESS correction, intra-run normalization, and linear model regression, was applied to eliminate the effect of GC bias [7, 8].

To determine if a tested maternal plasma sample belonged to a trisomy pregnancy, we calculated the *z*-score of % corresponding chromosome of the tested sample. The *z*-score is related to the number of standard deviations from the mean of a reference data set. The fetuses with trisomy 21, 18, 13, or sex chromosome aneuploidies were identified by Z score calculation using CapitalBio Data Analysis & Management Software BES 4000 Software as previously reported [9]. A Z score > 3 and fetal fraction ≥4% was set as the cutoff value to express the ratio of chromosome 21, 18, or 13 was increased, resulted from fetal trisomy 21, 18, or 13. Moreover, a Stouffer’s Z-score method was utilized to detect subchromosomal abnormalities more precisely with the combined Z scores in each 1 Mb region as report [10], in which, if the z score > 5, it was considered as microduplication, reversely if < − 5, microdeletion.

### Follow-up

All the participants were registered. If the NIPT results suggested trisomy 21, 18, 13, or sex chromosome aneuploidies, the amniocentesis was recommended for copy number variation (CNV) analysis; and the participants were followed up. If the CNV results showed abnormal, the karyotype analysis and/or microarray analysis were further recommended. The pregnant women who showed any NIPT abnormalities were classified as NIPT (+) or high-risk NIP group. For NIPT (+) pregnant women who refuse amniocentesis, the pregnancy was followed up for 1 year until the baby is delivered. For NIPT (−) pregnant women, karyotype analysis was based on their clinical indications and the participants’ willingness.

The specificity of the NIPT testing was evaluated by a questionnaire survey on the newborns’ health status. Newborns with no abnormalities in postpartum neonatal examinations were considered aneuploidy negative. Regardless of the NIPT results, incomplete pregnancy (miscarriage, stillbirth, and labor induction) and cases without a genetic diagnosis were not included in the NIPT test performance calculation.

### Statistical analysis

The statistical software SPSS 21.0 was used for statistical analysis of the descriptive data. The count data were expressed in the number of cases, frequency, and composition ratio. The comparison between groups was performed by χ^2^ test, and the difference was statistically significant with *P* < 0.05. The positive predictive value (PPV) was used to evaluate the performance of NIPT.

## Results

### The maternal age & gestational age distribution of pregnant women

**Fig. 1:**
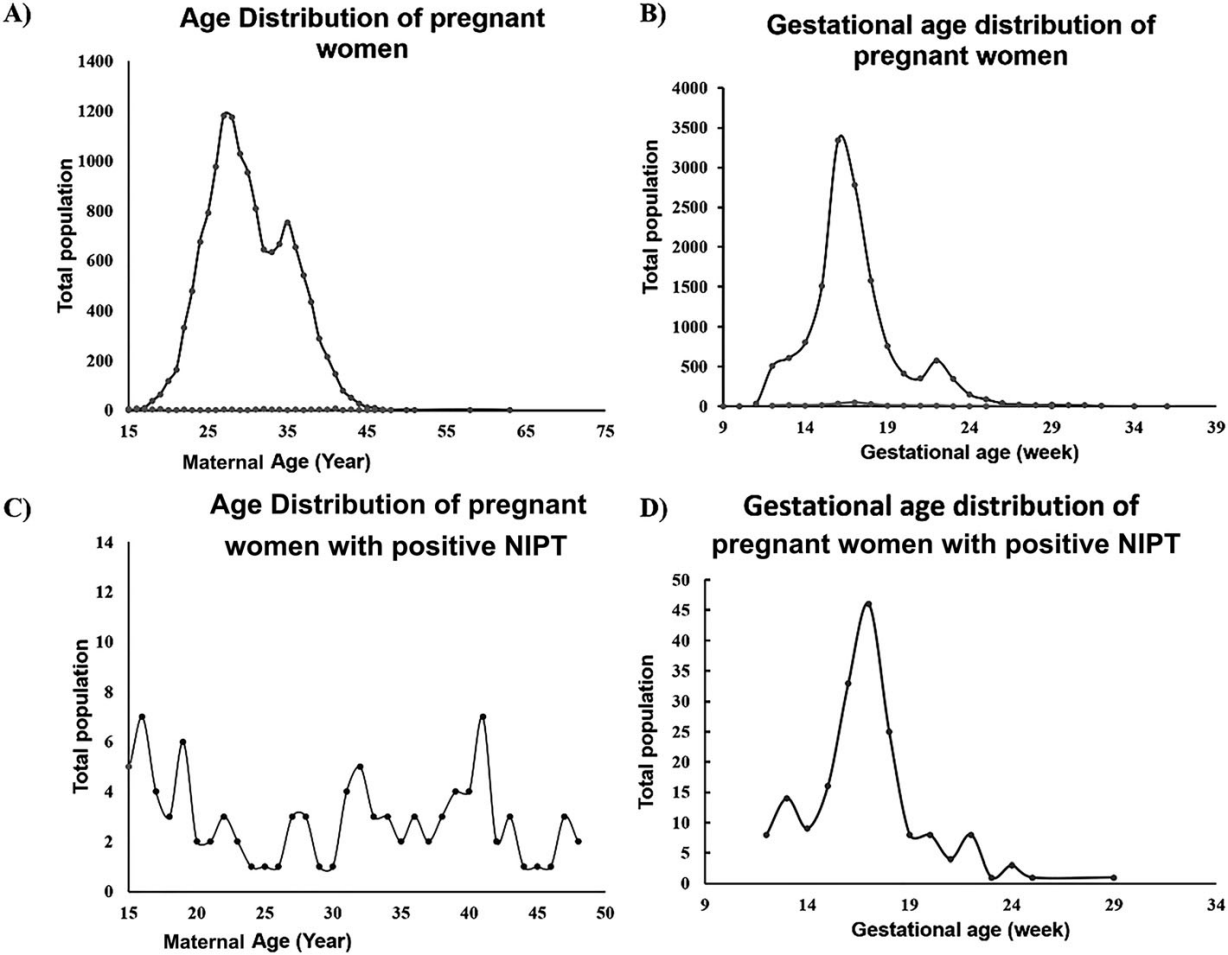
Maternal age and gestational age distribution of pregnant women. The black dots represent the population of pregnant women (y-axis) plotted against the corresponding maternal age(A/C), gestational age(B/D) (x-axis)

The pregnant women were divided into eight age groups with 5-year intervals. The number of pregnant women in each different age group is shown in Fig. 1A. The data showed that the main force of pregnant women giving childbirth is between 25 and 40 years old in Central China; the positive detection cases are also concentrated in this age group, which occupied 52.43% (97/185) of all positive cases (Fig. 1C). The time for the most commonly received examination was at 16 and 22 weeks of gestation. The gestational age distribution of the positive cases was shown in Fig. 1B and D.

### The relationship of cell-free fetal DNA concentration with gestational week and Z scores of trisomy 21, 18, 13

**Fig. 2:**
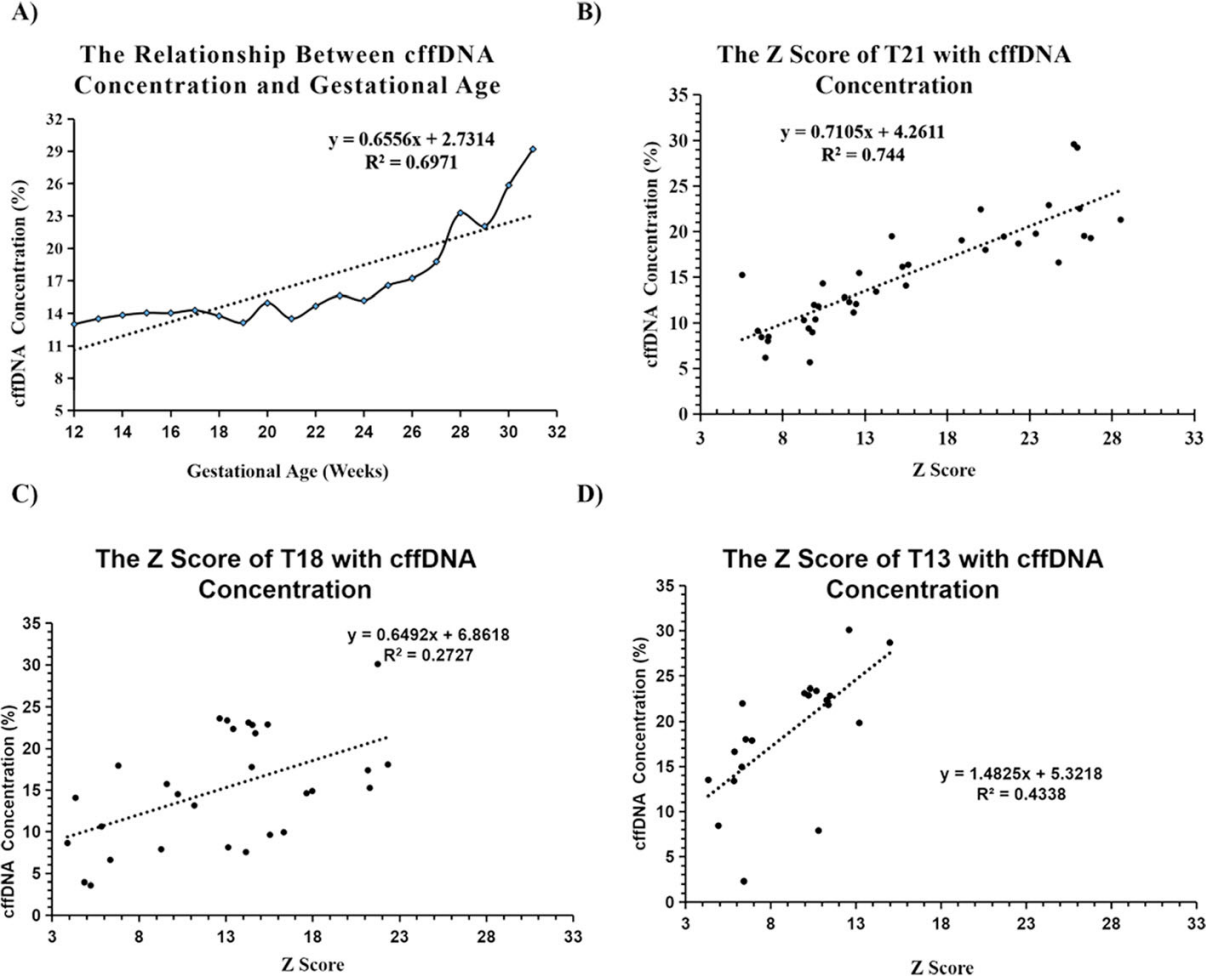
The relationship of estimated cffDNA concentration with gestational week and Z scores of trisomy 21, 18, 13. The black dots represent the cffDNA concentrations (y-axis) plotted against the corresponding gestational week (x-axis) and Z scores of trisomy 21, 18, 13

The cffDNA concentration was the well-known key factor in fetus aneuploidy detection [11]. The cffDNA concentration and its relationship with gestational week and the Z scores were further examined. We measured the cffDNA from 5656 plasma samples and correlated the cffDNA concentration with Z scores from nearly 80 plasma samples with positive NIPT for trisomy 21, 18, 13. The result showed that the amount of cffDNA increased significantly with the gestational week (Fig. 2A). They have a significant positive correlation; however, the coefficient rate was only 0.6971, which indicated that gestational week combined with other mechanisms might be responsible for driving the cffDNA concentrations. The correlation of the cffDNA concentration with the Z scores of positive NIPT further explores that cffDNA increased significantly with the Z scores of trisomy 21, 18, 13. There was a significant positive correlation with a coefficient of 0.744, 0.2727, and 0.4338, respectively (Fig. 2).

### Aneuploidy detection with NIPT test

A total of 189 cases of positive NIPT with chromosomal abnormalities were found in 14,316 pregnant women (Table 2). There was no significant difference in the positive detection rate between a single pregnancy and twin pregnancy (183/13,162 vs. 6/458, *p* > 0.05).

**Table 2:** The performance of NIPT for detection of fetal aneuploidy

NIPT result (*N* = 14,316)	Positive(n)	TP(n)	FN(n)	Sensitivity	Specificity	PPV	NPV
T21	49	45	1	97.83%	99.97%	91.84%	99.99%
T18	16	11	0	100.00%	99.99%	68.75%	100.0%
T13	16	6	0	100.00%	99.96%	37.50%	100.0%
XO	29	22	0	100.00%	99.96%	75.86%	NA
XXY	16	8	0	100.00%	99.96%	50.00%	NA
XYY	9	5	0	100.00%	99.98%	55.56%	NA
XXX	9	3	0	100.00%	99.96%	33.33%	NA
CNV	14	2	NA	–	–	14.29%	NA
other autosomal aneuploidy abnormalities	31	2	NA	–	–	6.45%	NA
Total	189	104		–	–	55.03%	NA

Since sex chromosome abnormalities and CNV abnormalities may not appear clinically during follow-up, it is impossible to calculate false negatives or false positives, so sensitivity and specificity are not calculated

*NPV* negative predictive value, *PPV* positive predictive value, *Positive* the NIPT(+), *TP* true positive, both NIPT(+) and interventional prenatal diagnosis (+), *FN* false negative, *PPV* positive predictive value, *NPV* negative predictive value

#### Trisomy 21, 18, 13 detections with NIPT test

The 49 pregnant women showed a T21 positive (0.34%), of which T13 accompanied one pregnant woman. The 48 patients (97.96%, 48/49) underwent interventional prenatal diagnosis, and 45 were diagnosed. Further analysis of 3 false-positive cases found that their Z scores were < 5, judged as a gray area according to the manufacturer’s instructions. In the confirmed cases, 2 cases had a Z value of < 5, and the amniocentesis showed a trisomy 21 mosaic karyotype (47, XN, + 21 [5]/ 46, XN [110] and 47, XN, + 21 [2]/ 46, XN [36], respectively). Also, there was one case in which a T21 diagnosis was missed. The pregnant woman was recalled for abnormal ultrasound screening and underwent a prenatal diagnosis for T21. The peripheral blood of this pregnant woman was collected and underwent NIPT. The result still showed a negative result; the possible explanation is that the maternal placenta might not coincide with fetal genetic information.

NIPT reported 16 patients with a T18 positive (0.11%), 13 of them (81.25%, 13/16) underwent prenatal diagnosis. Out of those, 11 patients were diagnosed, and 2 patients had false-positive cases with a Z value < 5. Three patients had no interventional prenatal diagnosis, one patient had multiple tumors in the fetus, one underwent an abortion, and one had a miscarriage.

NIPT reported a T13 positive in 16 patients (0.11%). Twelve patients (75%, 12/16) underwent prenatal diagnosis, and 6 were diagnosed. Four patients had no interventional prenatal diagnosis. One patient had multiple malformations confirmed by B-ultrasound. The other three declined any form of confirmation and chose to continue the pregnancy. There were no abnormalities found in follow-up within 12 months, and no abnormalities were found within 6 months after birth. The statistical results are shown in Table 2.

Together, these data showed that NIPT’s positive predictive value for T21, T18, and T13 is 91.84, 68.75, and 37.5%, respectively (Table 2).

#### SCAs detection with NIPT test

NIPT reported sex chromosome aneuploidy (SCA) abnormalities in 63 patients (0.44%), including 29 cases of 45 X, 16 cases of 47 XXY, 9 cases of 47 XXX, and 9 cases of 47 XYY. Forty-four patients (69.84%, 44/63) underwent prenatal diagnosis and 42 cases were confirmed (19 X monomers, 3 partial X monomers, 8 cases of 47 XXY, 2 cases of 47 XXX, 1 case 47 XXX/ 46 XX chimera, and 5 cases of 47 XYY). Two false-positive cases were due to the mother’s abnormality (47, XXX).

The PPV of NIPT for abnormal sex chromosome number is 66.67% (42/63). Among them, XO had the highest PPV of 75.86% (22/29), followed by XYY and XXY (55.56 and 50.0%, respectively) and XXX lowest (33.33%).

#### Other autosomal aneuploidies and CNV abnormalities

NIPT reported 31 cases (0.22%) of other uncommon autosomal aneuploidy abnormalities involving aneuploidy changes on chromosomes 3, 7, 8, 9, 10, 14, 15, 16, 20, and 22. Fourteen cases were confirmed CNV abnormalities (0.10%, 14/14, 316), involving CNV changes on chromosomes 4, 5, 7, 8, 10, 11, 14, 15, 16, and 22. A total of 19 cases (42.22%,19/45) received the invasive prenatal diagnosis, and karyotype analysis, 5 cases including one reported with T10, one reported with T8, one reported with microdeletion of chromosome 22, and one reported with microdeletion of chromosome 5 were confirmed. In the case of the microdeletion of chromosome 22, it was found that the pregnant woman did not declare that she was a balanced translocation carrier.

Among these cases, one pregnancy with 47 XN, + 7 and one pregnancy with microduplication of chromosome 12 ended in miscarriage. One woman who had two previous deliveries but had a chromosome 4 microdeletion chose to terminate the pregnancy. Other pregnant women declined any form of confirmation and chose to continue the pregnancy. There were no abnormalities that were observed in the follow-up within 12 months. In summary, the positive predictive value (PPV) of NIPT for other autosomal aneuploidy abnormalities was 6.45%. The PPV for CNV abnormalities was 14.29%.

### Prenatal indications of NIPT and test results

The clinical indications of NIPT in 14,316 pregnant women are shown in Table 1. Abnormal maternal serum screening (aMSS) group was the most common indication, accounting for 53.14%, followed by the advanced maternal age (AMA) group (23.02%) and voluntary testing group (17.63%). The thickened NT group’s positive detection rate was the highest (4.17%), and the voluntary testing group (3.49%) was the second. NIPT has different detection abilities in different indication groups and in statistically different positive cases of the indication group. The PPV of the twin/IVF-ET pregnancy was the highest, followed by the thickened NT group, the PPV value of other abnormal ultrasound abnormality on the fetus and placenta was the lowest. The lowest value may be associated with fewer positive cases and maternal refusion of prenatal diagnosis. The detection rate of T21, T18, T13, and sex chromosome abnormalities were similar in different indication groups without significant difference (*p* > 0.05).

## Discussion

The clinical significance of NIPT has been confirmed by several large-scale clinical studies [12, 13]. Of the 14,316 pregnant women in our study, NIPT results indicated that the high-risk T21/T18/T13 cases were 49 (0.34%), 16 (0.11%), and 16 (0.11%), respectively. The incomplete pregnancy (miscarriage, stillbirth, and labor induction) and cases without a genetic diagnosis were excluded, the other high-risk T21/T18/T13 cases were conducted invasive diagnosis confirmation or postnatal follow-up, the s true positive (TP) was 45 (0.31%), 11 (0.08%) and 6 (0.04%). NIPT PPV for the 21, 18, and 13-tris were 91.84, 68.75, and 37.5%, respectively. Our data is compatible with the other reported in China [14–16].

The relationship between the Z-score of high-risk T21/T18/T13 with NIPT’s accuracy was analyzed and showed that the relationship between cffDNA concentration and Z-score of high-risk T21/T18/T13 and gestational age was linear. The greater the gestational age (≥10 gestational weeks), the higher the cffDNA concentration and the positive specimen’s higher Z-score. The Z value of the false-positive cases of T13, T18, and T21 was also retrospectively analyzed. The false-positive cases were mainly with the Z < 5, which belonged to the gray area of the kit manual. This data further suggests that the Z score for NIPT testing is closely related to noninvasive prenatal testing accuracy.

Our data together indicated that clinical indications for NIPT testing should be strictly controlled. For NIPT results of 3 ≤ Z < 5, more appropriate post-test genetic counseling and further prenatal diagnostic guidance should be given. Also, studies have shown that low concentrations of cffDNA, confined placental mosaicism (CPM), vanishing twins, placental chimerism, and a low proportion of fetal chimerism may have false positives of 3 ≤ Z < 5 [17]. Therefore, it is strongly recommended that the combination of chromosomal karyotypes and microarray analysis for this group of pregnant women avoids misdiagnosis.

In this study, we found that the maternal age in central China is between 25 to 40, of which pregnant women aged over 35 years old counted for 23.02%. The advanced maternal age group’s NIPT positive rate was significantly higher than the serological high risk/critical risk group (chi-square = 74.04, *p* < 0.01). The positive detection rate of T21/T18/T13 was 1.12% (37/3295), indicating that at least 98.09% of advanced maternal age *pregnancies* can avoid invasive and risky *prenatal diagnosis* through NIPT. We further analyzed the proportion of advanced maternal age *pregnancies* before and *after imposing the “Two-Children” policy on January 1, 2016* [8, 9, 13]. We found that advanced maternal age pregnancies decreased significantly from 30.49% (182/597) to 22.69% (3113/13719) (Chi-square = 19.62, *p* < 0.01), indicating that the fertility desires of advanced maternal age are reduced due to high pregnancy complications and comorbidities. NIPT’s wide application may help alleviate this group’s concerns and increase fertility desires by improving the quality of birth to some extent.

The voluntary testing group counted for 17.63% of all pregnant women. This group’s positive detection rate is lower than the NT thickening group, probably due to public awareness of NIPT in pregnant women and the preferential policies and subsidies for NIPT from the government, which is critical for reducing congenital disabilities in Central China. Our data suggest that the NT thickening group’s positive detection rate and the true positive rate are higher than other indication groups, implying that NT thickening is riskier than other indication groups. Some experts recommend that such pregnant women should be directly counseled on prenatal diagnosis [18, 19]. We recommend that pregnant women should undergo prenatal testing for chromosomal aneuploidy if there are no contraindications. NIPT screening was also included for the patients with interventional surgery contraindication includes infection and coagulopathy in our study. However, our data didn’t show any significant difference owing to the low sample number. Our data support the reports that women infected with hepatitis B, hepatitis C, and/or human immunodeficiency virus (HIV) are recommended to use the noninvasive methods of prenatal risk assessment [8, 9, 13]; Also, it prefers to use the methods with high sensitivity and low false-positive rates, such as serum screening combined (or not) with nuchal translucency, anatomic ultrasound, and noninvasive molecular prenatal testing [5].

This study found that the overall positive predictive value of NIPT for sex chromosome abnormalities was 60.32%. The detection rate of aneuploidy, especially X+ type, was higher, which may be related to the pregnant women’s ovarian microenvironment at their ages. The accumulation of various harmful substances in the external environment increases the probability that the autosomes and sex chromosomes do not separate, which results in the increased incidence of fetal chromosomal abnormalities. The detection rate of sex chromosome abnormalities in this study was 60.32% (38/63), much lower than the detection rate of T21/T18/T13. It may be related to the mother X chromosome CNV [18], placental mosaic, or CPM. Besides, the mother’s X chromosome CNV may also cause X-linked disease in pregnancies with male fetuses, and it is necessary to conduct interventional tests.

We did not observe the difference of aneuploidy between single pregnancy and twin pregnancy, although it has reports mentioned that twin pregnancies may result in low fetal DNA concentration and a high failure rate of NIPT detection [19]. The results may be owing to the relatively small twin number versus huge single pregnancies in our study. Also, all 5656 plasma samples used for cffDNA concentration measurement were singleton pregnancies in our study; only singleton pregnancies were included in the statistical analysis of the relationship between the fetal concentration and the Z value of the positive result.

In this study, the twins and IVF pregnancies were classified in the same group; this is because the IVF pregnancies usually have a high incidence of multiple births, but they often choose to reduce their fetuses to singletons first trimester. On the other hand, if the pregnant woman carries a twin in China, it is regarded as precious fetuses with special attention and care by the whole family. However, both situations have a high detection failure rate; therefore, it is classified into the same group.

The significant factors contributing to false-positive and false-negative NIPT results were maternal copy number variant, vanishing twins, and fetal/placental mosaicism, but fetal fraction did not affect [20]. This study found 2 cases of 47 XXX pregnant women with sex chromosome abnormality in NIPT test results, indicating that the sensitivity and specificity of NIPT detection of SCAs are significantly lower compared with the typical T21/T18/T13. Some experts suggest combining maternal-plasma sequencing with maternal-WBC sequencing to avoid unnecessary invasive prenatal tests, assess the risk of X-linked disease to the fetus, and significantly improve NIPT’s accuracy for ChrX and ChrY [5]. Nevertheless, there are various biological reasons to consider, including maternal SCA mosaicism [18], vanishing twins, fetal under- or over-masculinization, CPM, or a maternal solid organ transplant [21] explain the discordance of NIPT sex chromosome result with that of sonographic or karyotype assessments.

A total of 31 cases of non-21/18/13 autosomal aneuploidy abnormalities and 14 cases of CNV abnormalities were detected in this study. Through the follow-up diagnosis of some patients with late follow-up and some positive patients, it was found that the current sequencing depth of NIPT and conventional algorithms could not apply to other routines. The abnormalities of NIPT results may not distinguish normal fetuses from other non-21/18/13 autosomal abnormalities. It must be noted that placental but not fetal DNA is examined in NIPT, and placenta genetic aberrations might be different from the fetus, which will lead to false positives. It is currently found that most pregnant women with non-21/18/13 chromosomal abnormalities in NIPT have a slow fetal development, and reasons remain to be further studied. In combination with current domestic and international expert consensus and guidelines, NIPT is not recommended for screening genome-wide CNV abnormalities [22–26]. Clinically, CNV-positive NIPT results should be treated with caution.

As a powerful screening approach, NIPT has dramatically changed the current status of prenatal screening. With the popularity of NIPT technology in low-risk pregnancy populations [20] and the development of high-throughput sequencing technologies, the beneficiaries of NIPT testing and the range of disease detection are also expanding [27, 28]. NIPT by whole genome sequencing often obtains positive results, which can link with biological explanation for the abnormalities from the fetal, and/or the placenta, and/or the pregnant woman. More clinical attention should be paid to genetic counseling and prenatal diagnosis before further clinical testing.

## Conclusion

Our data indicate that whole-genome sequencing of cffDNA has exceptionally high sensitivity and specificity for T21 and has a high sensitivity for T18, sex chromosome abnormalities, and T13. NIPT by whole-genome sequencing also provides evidence for other abnormal chromosomal karyotypes (CNV and non-21/18/13 autosomal aneuploidy abnormalities). The cffDNA concentration is closely related to the gestational age and determines the specificity of NIPT. Our results highlight the clinical significance for whole-genome sequencing of cffDNA as a useful prenatal screening approach for high-quality care of pregnancy.

## Data Availability

All data supporting the results reported in a published article can be found.

## Abbreviations

cffDNA: Cell-free fetal DNA
NIPT: Noninvasive prenatal testing
CNV: Copy number variation
NT: Nuchal translucency
CPM: Confined placental mosaicism
SCAs: Sex chromosome aneuploidy
AMA: Advanced maternal age
aMSS: Abnormal maternal serum screening; abnormally
aUS: Abnormal ultrasound findings
BMI: Body mass index

## References

[1] Alberry M, Maddocks D, Jones M, Abdel Hadi M, Abdel-Fattah S, Avent N, Soothill PW. Free fetal DNA in maternal plasma in anembryonic pregnancies: confirmation that the origin is the trophoblast. Prenatal diagnosis. 2007;27:415–8. 10.1002/pd.1700 17286310

[2] Cernat A, De Freitas C, Majid U, Trivedi F, Higgins C, Vanstone M (2019). Facilitating informed choice about non-invasive prenatal testing (NIPT): a systematic review and qualitative meta-synthesis of women’s experiences. BMC Pregnancy Childbirth.

[3] Pös O, Budiš J, Szemes T (2019). Recent trends in prenatal genetic screening and testing. F1000Res.

[4] Bayindir B, Dehaspe L, Brison N, Brady P, Ardui S, Kammoun M, Van der Veken L, Lichtenbelt K, Van den Bogaert K, Van Houdt J (2015). Noninvasive prenatal testing using a novel analysis pipeline to screen for all autosomal fetal aneuploidies improves pregnancy management. Eur J Hum Genet.

[5] Wang Y, Chen Y, Tian F, Zhang J, Song Z, Wu Y, Han X, Hu W, Ma D, Cram D (2014). Maternal mosaicism is a significant contributor to discordant sex chromosomal aneuploidies associated with noninvasive prenatal testing. Clin Chem.

[6] Yu T, Li S, Zhao W, Yu D (2019). False positive non-invasive prenatal testing results due to vanishing twins. Chin J Med Genet.

[7] Chen EZ, Chiu RWK, Sun H, Akolekar R, Chan KCA, Leung TY, Jiang P, Zheng YWL, Lun FMF, Chan LYS, Jin Y, Go ATJI, Lau ET, To WWK, Leung WC, Tang RYK, Au-Yeung SKC, Lam H, Kung YY, Zhang X, van Vugt JMG, Minekawa R, Tang MHY, Wang J, Oudejans CBM, Lau TK, Nicolaides KH, Lo YMD (2011). Noninvasive prenatal diagnosis of fetal trisomy 18 and trisomy 13 by maternal plasma DNA sequencing. PLoS One.

[8] Jiang F, Ren J, Chen F, Zhou Y, Xie J, Dan S, Su Y, Xie J, Yin B, Su W, Zhang H, Wang W, Chai X, Lin L, Guo H, Li Q, Li P, Yuan Y, Pan X, Li Y, Liu L, Chen H, Xuan Z, Chen S, Zhang C, Zhang H, Tian Z, Zhang Z, Jiang H, Zhao L, Zheng W, Li S, Li Y, Wang J, Wang J, Zhang X (2012). Noninvasive Fetal Trisomy (NIFTY) test: an advanced noninvasive prenatal diagnosis methodology for fetal autosomal and sex chromosomal aneuploidies. BMC Med Genet.

[9] Chiu RW, Chan KC, Gao Y, Lau VY, Zheng W, Leung TY, Foo CH, Xie B, Tsui NB, Lun FM (2008). Noninvasive prenatal diagnosis of fetal chromosomal aneuploidy by massively parallel genomic sequencing of DNA in maternal plasma. Proc Natl Acad Sci U S A.

[10] Yin A-h, Peng C-f, Zhao X, Caughey BA, Yang J-x, Liu J, Huang W-w, Liu C, Luo D-h, Liu H-I (2015). Noninvasive detection of fetal subchromosomal abnormalities by semiconductor sequencing of maternal plasma DNA. Proc Natl Acad Sci U S A.

[11] Illanes S, Denbow M, Kailasam C, Finning K, Soothill PW (2007). Early detection of cell-free fetal DNA in maternal plasma. Early Hum Dev.

[12] Gil MM, Accurti V, Santacruz B, Plana MN, Nicolaides KH (2017). Analysis of cell-free DNA in maternal blood in screening for aneuploidies: updated meta-analysis. Ultrasound Obstet Gynecol.

[13] Taylor-Phillips S, Freeman K, Geppert J, Agbebiyi A, Uthman OA, Madan J, Clarke A, Quenby S, Clarke A (2016). Accuracy of non-invasive prenatal testing using cell-free DNA for detection of down, Edwards and Patau syndromes: a systematic review and meta-analysis. BMJ Open.

[14] Weiying M, Peng Z, Youqiong L, Yaoxi M, Hongyan Z, Renfeng Z (2018). Application of non-invasive DNA prenatal diagnosis for pregnant women with advanced maternal age. Chin J Clin Obstet Gynecol.

[15] Ying L, Ping H, Dingyuan M, Fuman J, Xiuqing J, An L, Jian C, Jingjing Z, Jing Z, Tao J (2014). Analysis of the results of non-invasive genetic testing for common fetal chromosomal aneuploidy and genetic counseling. Chin J Med Genet.

[16] Honglei D, Jie L, Yuan X, Yali H (2014). Clinical indications and detection efficiency of non-invasive prenatal testing in 13 041 cases from Jiangsu. Chin J Perinat Med.

[17] Tian Y, Zhang L, Tian W, Gao J, Jia L, Cui S (2018). Analysis of the accuracy of Z-scores of non-invasive prenatal testing for fetal Trisomies 13, 18, and 21 that employs the ion proton semiconductor sequencing platform. Mol Cytogenet.

[18] Wang S, Huang S, Ma L, Liang L, Zhang J, Zhang J, Cram DS (2015). Maternal X chromosome copy number variations are associated with discordant fetal sex chromosome aneuploidies detected by noninvasive prenatal testing. Clin Chim Acta.

[19] Gil MM, Galeva S, Jani J, Konstantinidou L, Akolekar R, Plana MN, Nicolaides KH (2019). Screening for trisomies by cfDNA testing of maternal blood in twin pregnancy: update of the Fetal Medicine Foundation results and meta-analysis. Ultrasound Obstet Gynecol.

[20] Zhang H, Gao Y, Jiang F, Fu M, Yuan Y, Guo Y, Zhu Z, Lin M, Liu Q, Tian Z (2015). Non-invasive prenatal testing for trisomies 21, 18 and 13: clinical experience from 146 958 pregnancies. Ultrasound Obstet Gynecol.

[21] Bianchi D, Swanson A, Parsa S, Bhatt S, Halks-Miller M, Sehnert A, Rava R (2014). NIPT for sex chromosome aneuploidy: initial clinical laboratory experience and biologic reasons for discordant results.

[22] Grati FR (2016). Implications of fetoplacental mosaicism on cell-free DNA testing: a review of a common biological phenomenon. Ultrasound Obstet Gynecol.

[23] Gregg AR, Skotko BG, Benkendorf JL, Monaghan KG, Bajaj K, Best RG, Klugman S, Watson MS (2016). Noninvasive prenatal screening for fetal aneuploidy, 2016 update: a position statement of the American College of Medical Genetics and Genomics. Genet Med.

[24] Song Y, Huang S, Zhou X, Jiang Y, Qi Q, Bian X, Zhang J, Yan Y, Cram DS, Liu J. Non-invasive prenatal testing for fetal aneuploidies in the first trimester of pregnancy. Ultrasound Obstet Gynecol. 2015;45(1):55–60. 10.1002/uog.13460. 10.1002/uog.13460 25044397

[25] Devers PL, Cronister A, Ormond KE, Facio F, Brasington CK, Flodman P (2013). Noninvasive prenatal testing/noninvasive prenatal diagnosis: the position of the National Society of Genetic Counselors. J Genet Couns.

[26] Salomon LJ, Alfirevic Z, Audibert F, Kagan KO, Paladini D, Yeo G, Raine-Fenning N (2014). ISUOG consensus statement on the impact of non-invasive prenatal testing (NIPT) on prenatal ultrasound practice. Ultrasound Obstet Gynecol.

[27] Wapner RJ, Babiarz JE, Levy B, Stosic M, Zimmermann B, Sigurjonsson S, Wayham N, Ryan A, Banjevic M, Lacroute P (2015). Expanding the scope of noninvasive prenatal testing: detection of fetal microdeletion syndromes. Am J Obstet Gynecol.

[28] Jia Y, Zhao H, Shi D, Peng W, Xie L, Wang W, Jiang F, Zhang H, Wang X (2014). Genetic effects of a 13q31.1 microdeletion detected by noninvasive prenatal testing (NIPT). Int J Clin Exp Pathol.

